# Origin of the poxviral membrane: A 50-year-old riddle

**DOI:** 10.1371/journal.ppat.1007002

**Published:** 2018-06-21

**Authors:** Bernard Moss

**Affiliations:** Laboratory of Viral Diseases, National Institute of Allergy and Infectious Diseases, National Institutes of Health, Bethesda, Maryland, United States of America; University of Florida, UNITED STATES

Over the past half century, fundamental questions regarding the structure and origin of poxviral membranes have confounded researchers. Poxviruses are DNA viruses that infect vertebrates and invertebrates and are distantly related to other nucleocytoplasmic large DNA viruses (NCLDVs), including phycodnaviruses, iridoviruses, asfarviruses, mimiviruses, and additional giant viruses. The poxviruses replicate entirely within the cytoplasm, cause human and zoonotic diseases, and are useful vaccine vectors [1]. The brick-shaped infectious mature virion (MV) has a nucleoprotein core enclosed by a lipoprotein envelope and arises from the spherical immature virion (IV). During replication, the MVs of some poxviruses are wrapped with a second membrane that facilitates intracellular transport, exocytosis, and dissemination. The membrane of the MV is referred to here as an envelope and the second one as a wrapping membrane. This short review describes the convoluted path leading to our current understanding of the structure and formation of the poxvirus envelope, obtained mainly through studies of vaccinia virus (VACV), the prototype poxvirus. Readers interested in the wrapping membrane are referred to a recent article [2] and references therein that posit their formation from modified Golgi cisternae following retrograde transport of a key viral protein.

## A de novo origin of the poxviral envelope

**Fig 1:**
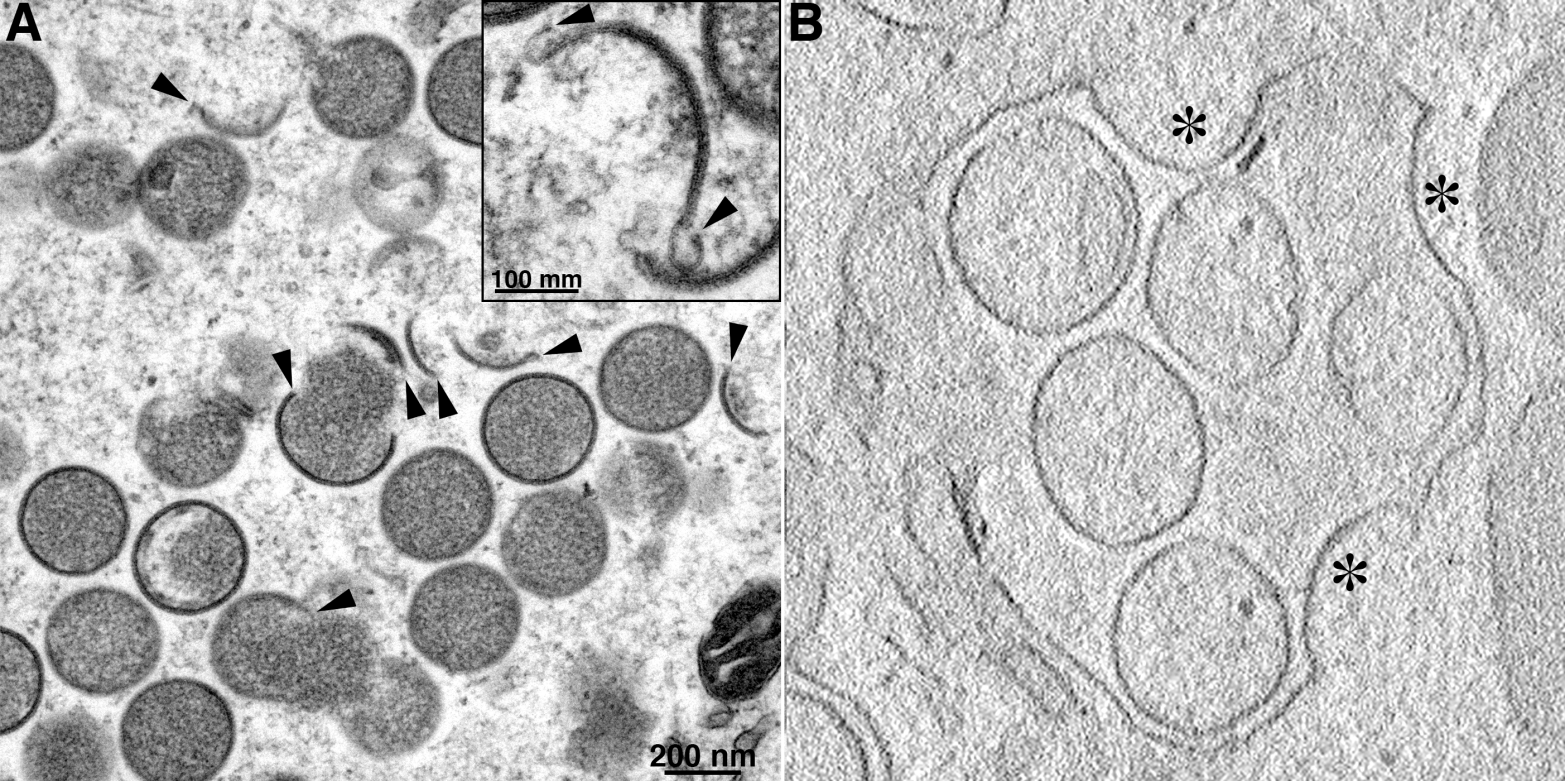
Transmission electron microscopic images of viral structures formed during infection with wild-type VACV and a VMAP (viral membrane assembly protein) mutant. (A) Infection with wild-type VACV. A cluster of crescents with free ends, some of which are marked with arrowheads, and circular IVs are shown. The higher magnification in the inset reveals the spicule layer on the convex surface of crescents as well as characteristic uncoated curls at their ends. (B) Infection with the VMAP A30.5 deletion mutant. This image is from an electron tomography tilt series that shows continuity between curved viral crescent structures, marked by asterisks, and the smooth ER membrane. The crescents appear to be budding into the expanded lumen, which is filled with IV-like structures. *Images kindly provided by A*. *Weisberg*.

The first unique structures that can be visualized by transmission electron microscopy of cells infected with VACV appear in the cytoplasmic factory areas as crescent membranes (cupules in three dimensions) and circular IVs (spherical in three dimensions) that partially or completely enclose electron-dense core proteins. In the images provided by Dales and Mosbach in 1968 [3] and a recent one shown in Fig 1A, the crescents appear to have free ends (or edges in three dimensions) and appear to be comprised of a single membrane bilayer coated with dense material sometimes resolved into spicules on the convex surface (Fig 1A, inset). The drug rifampicin interrupts morphogenesis resulting in the formation of single membranes that are irregular and lack the spicule coat [4]. Dales and Mosbach [3] stated, “…we have examined thousands of sectioned cell profiles containing developmental stages of the virus [VACV], but have never observed any morphological continuity between the viral envelopes and any of the adjacent cellular membranes. Therefore, on morphological grounds alone, we assumed that the vaccinia membranes are organized de novo.” This was a novel proposal because the envelopes of other viruses are derived from cellular organelles, and cellular membranes arise from pre-existing membranes. Although unprecedented, this hypothesis could not be summarily dismissed since the 200 or more genes of poxviruses encode all or nearly all of the proteins needed for transcription and replication and even for a unique cytoplasmic redox system used for disulfide bond formation [5]. Thus, there was a possibility that some yet uncharacterized poxvirus proteins enable de novo membrane assembly.

## An alternate hypothesis for poxviral envelope formation

More than 30 years later, in a review article entitled “Assembly of vaccinia virus revisited: de novo membrane synthesis or acquisition from the host” [6], the authors stated, “For a biochemist or a cell biologist this hypothesis [de novo membrane formation] is heretical.” Particularly, a single membrane bilayer with free ends in the cytoplasm was anathema. In constructing an alternative model in which the viral membrane is a closed cisterna, they cited studies suggesting that (i) the poxviral membrane is composed of 2 tightly apposed membranes without free ends, (ii) the spicule coat is on the concave surface of the viral crescent rather than the convex surface, (iii) the membrane is formed from the intermediate compartment of the secretory system (ERGIC) by a wrapping process, and (iv) the “double membrane” on MVs is discarded prior to entry and the core somehow transported across the plasma membrane to enter the cytoplasm by an unknown nonfusion mechanism. These conclusions provoked considerable controversy, and each was disproved by investigators who affirmed the single-bilayer structure of the membrane [7, 8], demonstrated that the spicule layer is a honeycomb lattice comprised of trimers of the D13 scaffold protein on the convex surface of the IV [8, 9], reported that transport of viral proteins to the intermediate compartment is not required for viral envelope formation [10], and showed that MV entry involves membrane fusion at the plasma or endosomal membrane [11, 12]. The controversy regarding the structure of the crescent membrane ended when the original proponents of the double membrane model, and their collaborators provided electron tomographic evidence for single-bilayer membranes with free ends referred to as open sheets [13, 14], essentially as depicted decades earlier by Dales and others [3, 4]. Nevertheless, the origin of the crescent membrane remained ambiguous.

## Connections between the endoplasmic reticulum (ER) and viral membranes revealed

Although circumstantial evidence—including the presence of ER membranes within factories and ER localization of some viral membrane proteins—suggested a role for the ER in forming the viral envelope, even state-of-the-art electron microscopic methods failed to demonstrate continuity of viral and cellular membranes. One possible explanation for this failure was that the junctions between the viral membrane and the putative cellular precursors were too fleeting for capture by electron microscopy during a normal infection [15]. An offshoot of this idea was that the connections might persist during abortive infection by a mutant virus with an assembly block. Five small viral proteins that are conserved in all poxviruses were found to be individually essential for VACV assembly and collectively called VMAPs (viral membrane assembly proteins) [16–20]. Remarkably, electron microscopic images of cells abortively infected with VMAP mutants (Fig 1B) revealed connections between spicule coated curved membranes and smooth membranes [20]. The curved membranes were shown by immunogold labeling to contain the major viral membrane protein A17 and the D13 scaffold protein, whereas the connecting smooth membranes contained the ER membrane protein calnexin [21, 22]. Furthermore, the connections between viral membranes and the ER were confirmed by electron tomography studies carried out with each of the VMAP deletion mutants [23]. The crescents bud into the expanded ER lumen where IV-like particles accumulate (Fig 1B), indicating that the inner leaflet of the ER forms the convex surface of these viral particles. The IV-like particles appear empty as the core proteins are deposited in large aggregates outside of the ER. Shedding of the scaffold layer and transformation of the spherical particles to the brick shape characteristic of MVs fails to occur. In addition, the hydrophobic protein components of the entry-fusion complex are unstable due to their failure to insert into the IV-like membranes. The finding of the D13 scaffold on the luminal side of the modified ER membrane suggests that some interruptions in the ER membrane provide access for the D13 protein. The frequency of such interruptions may vary in different cell lines and conditions, which would explain why the ER-associated crescents and IV-like particles are more abundant in some cells infected with VMAP mutants than others [21]. During an infection with wild-type VACV, the predilection of the D13 protein for the inner leaflet of the ER could ensure that crescent formation follows ER breakage.

Single-bilayer membranes with free ends have also been detected in the viral factory areas of cells infected with other members of the NCLDV, notably African swine fever virus and mimiviruses, suggesting that their envelopes may form from the ER by a mechanism similar to that of poxviruses [24–26]. While many unrelated RNA viruses also derive their envelopes from the ER, they do so by budding into the lumen resembling the IV-like particles that form with the VMAP mutants of VACV.

## A model for poxviral membrane biogenesis

**Fig 2:**
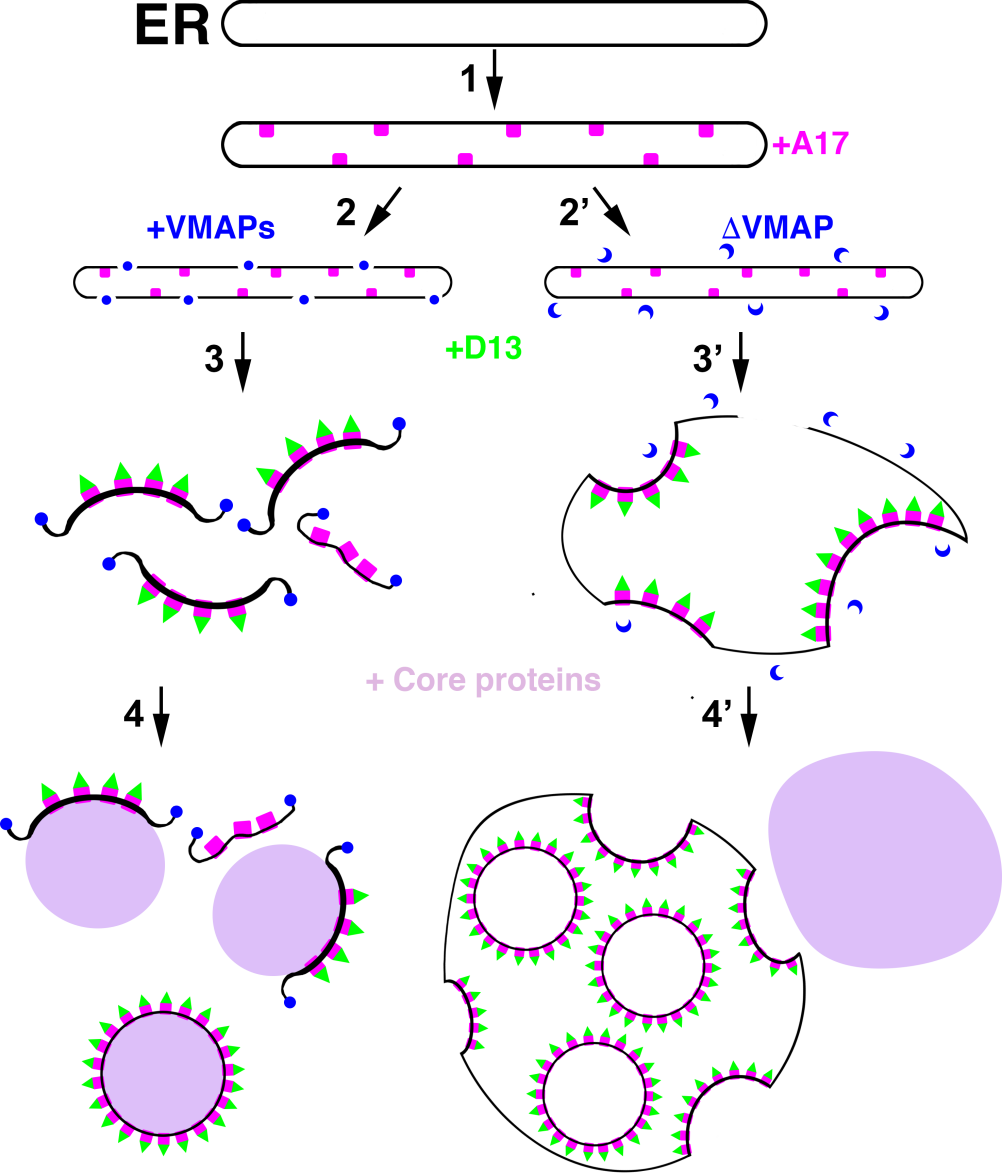
Model for poxviral membrane biogenesis. The first step (1) consists of modification of the ER by insertion of the A17 transmembrane protein represented by pink boxes. In the presence of all 5 VMAPs represented by blue spheres, membrane scission occurs, and the edges of the sheets are stabilized (2). D13 trimers, represented by green triangles, associate with the N-terminus of A17 to increase curvature forming crescent structures (3), which extend by fusion with additional membrane segments around core proteins (violet) to form the spherical IVs (4). VMAPs missing at least one component (ΔVMAP) represented by blue sickle shapes are unable to induce or stabilize membrane scissions (2’), the crescents remain attached to the ER (3’), and empty IV-like particles bud into the lumen while the core proteins form dense aggregates (violet) outside of the ER (4’). *Drawings kindly provided by A*. *Weisberg*.

In a provisional model of viral membrane formation (Fig 2), the first step is modification of the ER within the viral factory by insertion of the viral A17 and other integral membrane proteins. All 5 VMAPs, likely in concert with cellular proteins, participate in severing the modified ER and/or stabilizing breaks that normally occur transiently, as shown in Fig 2, step 2. Association of D13 trimers with A17 leads to the formation of curved crescents (Fig 2, step 3). The crescents enlarge by accretion of additional modified ER membrane and associate with core proteins to form IVs (Fig 2, step 4), which subsequently mature into infectious virus particles. The finding that VMAPs localize at the free ends of viral crescent membranes during a normal infection [21] and that IV-like particles associated with ER form when A17 and D13 are expressed in the absence of other viral late proteins [23] provides additional support for the model. An important feature of this model is that enveloped viral particles are free in the cytoplasm. In the absence of any one VMAP, continuity of the modified ER and viral membrane is largely retained during viral crescent formation (Fig 2, step 2’), although there are evidently some discontinuities that allow the D13 scaffold protein to enter the lumen. The crescents appear to bud into the lumen of the ER (Fig 2, step 3’) and form IV-like particles lacking core proteins (Fig 2, step 4’) that fail to mature into infectious virions.

The ER is a dynamic structure, and the molecular events that enable breakage is an intriguing topic for cell biologists as well as virologists. Although this model or some variation in which the poxviral envelope is derived from the ER, is well supported by the existing data and favored by the author, the possibility that viral proteins are diverted to the ER in the absence of VMAPs, resulting in an anomalous site of envelope formation cannot be ruled out. Thus, the saga of poxvirus membrane biogenesis continues.
